# Anatase-Free Nanosized Hierarchical Titanosilicate TS-1 Synthesis via Nitric Acid-Catalyzed Gel Preparation

**DOI:** 10.3390/gels11080605

**Published:** 2025-08-02

**Authors:** Vera R. Bikbaeva, Sergey V. Bubennov, Dmitry V. Serebrennikov, Daria A. Ogurechnikova, Evgenii V. Vakulin, Boris I. Kutepov, Nellia G. Grigoreva, Anton L. Maximov

**Affiliations:** 1 Institute of Petrochemistry and Catalysis of the Ufa Federal Research Center, The Russian Academy of Sciences, Ufa 450075, Russia; 2 Faculty of Technology, Ufa State Petroleum Technological University, Ufa 450064, Russia; 3 A.V. Topchiev Institute of Petrochemical Synthesis, Russian Academy of Sciences (TIPS RAS), Moscow 119991, Russia

**Keywords:** TS-1, titanosilicates, nanosized catalysts, hierarchical porous structure, cyclohexene oxidation, hydrogen peroxide, epoxidation, catalyst synthesis

## Abstract

A new approach to the synthesis of a nanosized and hierarchical titanosilicate, TS-1, is presented. Instead of using specific solid or additional mesoporous templates or individual additives to slow down the hydrolysis of titanium alkoxides, it is proposed that the titanosilicate TS-1 can be obtained from gels synthesized with hydrolysis catalysts (HNO_3_ and tetrapropylammonium hydroxide). When nitric acid catalyzes tetraethyl orthosilicate (TEOS) hydrolysis, the resulting crystalline TS-1 that can be obtained has uniform particle sizes (150–180 nm), is anatase-free, and contains up to 46–67% of mesopores. When a base catalyst is applied, the obtained material’s features are opposite. Moreover, acid-promoted TS-1 samples catalyze cyclohexene H_2_O_2_-oxidation via a heterolytic route to the cyclohexane epoxide with 67% selectivity, which is non-typical.

## 1. Introduction

Heteroatom zeolites are a subclass of microporous crystalline materials with a defined ordered structure. Titanosilicates are also included in this subclass, and their main advantages and disadvantages largely coincide with the corresponding characteristics of zeolites. The titanosilicate TS-1 is one of the most well-known materials with an MFI structure [1]. It is successfully used in industry as a catalyst in the HPPO process for the production of propylene oxide [2]. TS-1 has found widespread application in the development of new “green” methods for the oxidation of a wide range of alkenes [3], cyclohexanone ammoximation, the hydroxylation of phenol [4], and oxidative desulfurization [5].

Modern requirements [6,7] for new active catalytic systems based on titanosilicates imply the following quality criteria for the synthesized materials: nanosized particles, reduced diffusional limitations, complete incorporation of the metal into the crystalline silicate lattice, and the presence of active centers of specific types and strengths [8].

For the synthesis of TS-1, the following methods are commonly employed: hydrothermal synthesis, dry gel conversion, solvent-free synthesis, isomorphous substitution, and microwave-assisted synthesis. In the vast majority of cases, acids are not introduced at any stage of the synthesis. Final crystallization typically occurs at pH > 7, with rare exceptions where synthesis is carried out in the presence of excess halogen acids [9].

At the same time, reports from the literature indicate positive effects of introducing acids in catalytic amounts into systems containing sources of silicon and titanium. The hydrolysis of tetraethoxysilane proceeds rapidly in the presence of acids, while the subsequent condensation stage is slowed down. “Acidic” sols consist of linear polymers rather than spherical particles. Moreover, the terminal groups of growing chains exhibit enhanced reactivity [10]. Therefore, this approach may promote better incorporation of heteroatoms into the developing silicate structure. On the other hand, nitric acid, within a certain molar ratio range of H^+^/Ti, contributes to the stabilization of titanium-containing sols, and this technique (acid addition) is successfully used in the synthesis of titanium dioxide [11,12]. However, when the number of moles of acid exceeds that of titanium alkoxide, the latter precipitates immediately as a solid in aqueous-isopropanolic solutions and readily transforms into anatase or rutile without exposure to high temperatures. In such cases, the acidified alkoxide process results in the formation of smaller particles with a higher specific surface area [13]. Thus, there is reason to believe that during the synthesis of titanosilicates, the hydrolysis of titanium alkoxides can be initiated in the presence of catalytic amounts of inorganic acids (HCl, HNO_3_) to improve the incorporation of metal into the silicate framework.

The acids used for titanosilicate synthesis are generally applied for sample modification. For instance, the layered titanosilicate AM-4 could be modified by diluted nitric acid (up to 0.25 M) [14]. Such a method allows the exchange of sodium cations for protons in the interlayer space to upgrade activity. A similar method for increasing the catalytic activity of a material is described in previous works, [15] (diluted HF for ETS-10 molecular sieves) and [16] (H_2_SO_4_, HCl, and H_3_PO_4_ for silica-titania aerogel). More frequently, acids are used for the removal of extra-framework TiO_2_ without framework distraction. For the TS-1 material, this approach is described in [17]. Treatment over 24 h using solutions of nitric, sulfuric, and hydrochloric acids principally eliminate the extra-framework Ti.

Another approach to acid utilization during titanosilicate synthesis is the application of acid for titanium’s introduction into the framework (isomorphous substitution tetrahedral heteroatom).

There are only a few reported examples of the application of acids of various types at different stages of TS-1 molecular sieve synthesis and their influence on the physicochemical properties of the resulting materials. For instance, Ti-MOR zeolite may be synthesized by a combination of acid treatment and TiCl_4_ evaporation [18]. HNO_3_-modulated substitution of the ERB-1 zeolite allows the formation of a Ti-MWW heteroatomic zeolite [19].

Acids’ application for direct synthesis, rather than for the removal of extra-framework Ti or the dealumination of parent zeolite before Ti introduction, is not widespread. The use of amino acids, such as l-lysine, in the synthesis of crystalline titanosilicates promotes the formation of TiO_6_ units and their subsequent stabilization [20]. In this approach, the amino acid is added after the hydrolysis of tetraethyl orthosilicate (TEOS) and the titanium source, tetrabutyl orthotitanate (TBOT), is complete. The addition of L-lysine helps to lower the pH of the resulting gel, which in turn slows the hydrolysis of the titanium precursor and facilitates its incorporation into the framework. It has been demonstrated that the pH of the gel decreases from 12.1 (without lysine) to 11.1 at a molar ratio of Si/L-lysine = 0.4 [21]. Generally, both silicon and titanium sources are introduced under alkaline conditions.

The use of a fluorine-containing acid (HF) in the synthesis of titanosilicate TS-1 results in the formation of both very large particles (greater than 30 μm) and aggregates up to 30 nm in size [22]. According to [23], the introduction of hydrofluoric acid during the crystallization of TS-1 xerogels enables the production of crystallites smaller than 1 μm. Additionally, a method for synthesizing the titanosilicate TS-1 using 1,3,5-benzenetricarboxylic acid as a hydrolysis modifier has been reported [24]. In this method, a solution of the acid is added together with TPAOH to a mixture containing TEOS and tetrabutyl orthotitanate, which increases the number of nuclei and yields aggregated particles composed of nanocrystals.

The use of acids in the synthesis of amorphous porous titanosilicates has also been mentioned. For example, in a previous study [25], nitric acid was employed to obtain TiO(NO_3_)_2_ with the aim of subsequently incorporating it into the silicate matrix as isolated Ti^4+^ centers.

Summarizing the role of various acids in the synthesis of TiO_2_ and titanosilicates, it can be suggested that the presence of protons in the reaction mixture during the early stages of titanosilicate synthesis may facilitate improved incorporation of titanium into the crystalline structure, prevent self-condensation of titanium alkoxides, and reduce crystal size.

In light of the above, the objective of the present study is to develop a method for synthesizing a variant of the nanosized titanosilicate TS-1 with enhanced porosity and without extra-framework titanium, using limited amounts of nitric acid. The catalytic properties of the obtained TS-1 samples were investigated through the oxidation of cyclohexene with hydrogen peroxide in a methanol-free medium.

## 2. Results and Discussion

### 2.1. Physicochemical Properties of Titanosilicate Catalysts

The titanosilicates obtained in this work differed in their preparation method. To maximize the incorporation of framework titanium atoms into the TS-1 titanosilicate structure, the rate of hydrolysis of the silicon and titanium sources during the initial stage (formation of the precursor gel) was controlled by the introduction of a catalyst. The samples obtained when nitric acid solution was used as the hydrolysis catalyst are hereafter designated as TS-1-a. The samples acquired when a base (TPAOH) was used are designated as TS-1-b. The molar ratio of Si/H^+^ and Si/OH^−^ was maintained at 120:1. The second stage, i.e., the crystallization of the precursor gels formed under these conditions, was carried out in two steps under identical conditions for both samples: 24 h at 60 °C, followed by 72 h at 170 °C. The resulting samples are labeled as follows: TS-1-a-24/60-72/170 for the samples prepared with the addition of HNO_3_ acid, and TS-1-b-24/60-72/170 for the samples prepared with the addition of a base.

Since hydrothermal synthesis conditions (time and temperature) can significantly affect the properties of the resulting materials, a series of TS-1 samples was synthesized from the acid-containing gel with varying crystallization conditions. Hydrothermal synthesis of the acid-containing gel was performed either in a single step (24 h at 170 °C or 24 h at 130 °C) or in two steps (first step: 24 h at 60 °C; second step: 24 h at 130 °C or 170 °C). Samples of the first type are designated as TS-1-a-24/T, where T corresponds to the crystallization temperature (°C). Samples of the second type are designated as TS-1-a-24/T_1_-X/T_2_, where T_1_ is the aging temperature (°C), X is the crystallization time (h), and T_2_ is the crystallization temperature (°C).

#### 2.1.1. Structural Properties of Titanosilicates

**Figure 1:**
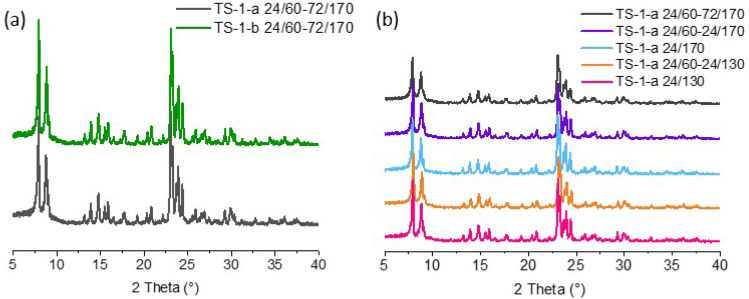
(**a**) XRD patterns of solids of TS-1 synthesized from acid- and base-catalyzed gels; (**b**) XRD patterns of TS-1 synthesized from acid-catalyzed gels under variated hydrothermal treatment conditions.

Figure 1 shows the diffraction patterns of the obtained samples.

The presence of peaks 2θ = 7.95, 8.84, 23.10, 23.95, 24.4° indicates the formation of MFI topology. The diffraction peaks are characterized by high intensity. All samples have a high degree of crystallinity. The presence on the X-ray diffraction patterns of diffraction peaks only corresponding to the MFI zeolite with high phase purity and crystallinity indicates the absence of new phase formation after the use of nitric acid at the precursor gel preparation stage. The amorphous phase is absent in the materials. The obtained results show that changing both the conditions of the precursor gel synthesis and the conditions of hydrothermal crystallization (time, temperature) allows the acquirement of phase-pure materials with a crystalline structure.

#### 2.1.2. Morphology of Titanosilicates

**Figure 2:**
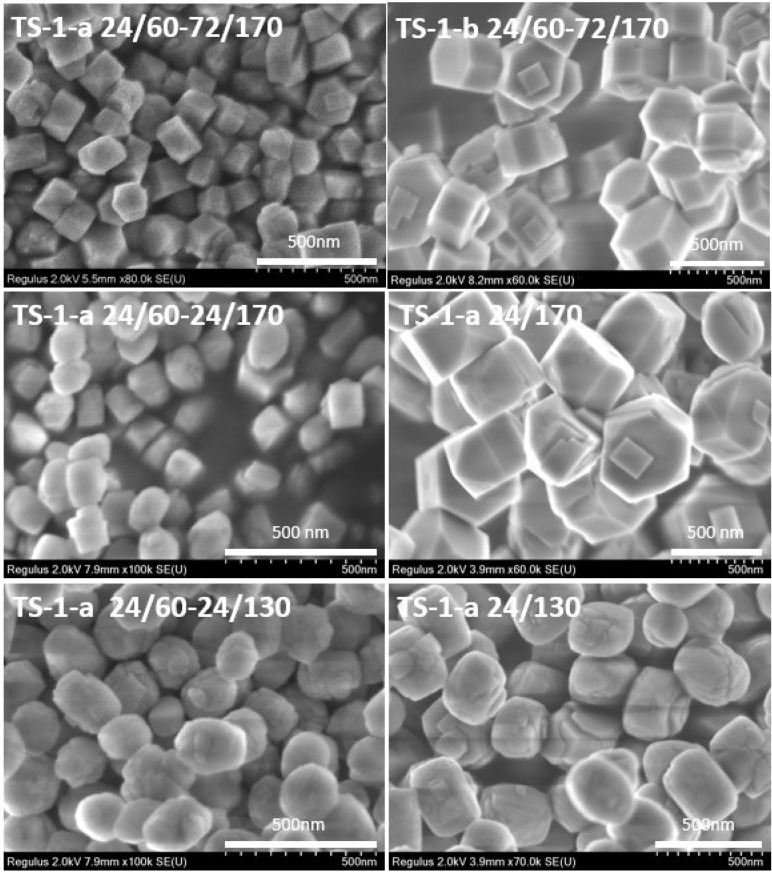
SEM images of samples synthesized from acid- and base-catalyzed gels under varying crystallization conditions.

SEM image analysis (Figure 2) revealed that the synthesis method has a significant influence on the morphology and size of TS-1 crystals.

Crystals of the TS-1-a samples (obtained by crystallization of an acid-containing precursor gel) are more elongated than the crystals of the TS-1-b titanosilicate (obtained from a precursor gel synthesized with the base addition during the TEOS hydrolysis step). Furthermore, the method of preparing the precursor gel affects the average crystal size of TS-1 titanosilicate synthesized under identical hydrothermal crystallization conditions (24/60–24/170): TS-1-a crystals are smaller than TS-1-b crystals (200–220 nm and 330–370 nm, respectively).

In single-step crystallization of the acid-containing gel, lowering the process temperature from 170 °C to 130 °C results in smaller crystals (480–550 nm for the TS-1-a 24/170 sample and 300–380 nm for the TS-1-a 24/130 sample).

When crystallization of the acid-containing gel is carried out in two stages, the smallest crystals are formed (150–180 nm), with the temperature of the second stage (130 °C or 170 °C) having little effect on crystallite size. Decreasing the crystallization temperature at the second stage from 170 °C to 130 °C promotes a morphological change from well-defined hexagonal prisms to more rounded elliptical forms, as well as the formation of flake-like nanoaggregates on the surface. The size of these flake-like aggregates is 40–80 nm for the TS-1-a 24/60–24/130 sample. For the TS-1-a 24/60–24/170 sample, a low amount of nanocrystals that are approximately 50 nm in size are observed.

Increasing the time of the second stage of crystallization from 24 h to 72 h for TS-1-a samples leads to an increase in the crystal sizes from 150–180 nm to 200–220 nm. At the same time, for the TS-1-a 24/60-72/170 sample, the presence of small crystallites on the surface of the main crystal is practically not detected, and the average thickness of the crystallites is 120 nm.

Thus, the average particle size (excluding flocculent aggregates) decreases in the following order: 480–550 nm (TS-1-a 24/170) > 380–350 nm (TS-1-a 24/130) > 370–330 nm (TS-1-b 24/60-72/170) > 220–200 nm (TS-1-a 24/60-72/170) > 180–150 nm (TS-1-a 24/60-24/170) ≈ 180–150 nm (TS-1-a 24/60-24/130). Titanosilicates with the largest particles of regular hexagonal shape, approximately 550 nm in size, were obtained by a one-step high-temperature crystallization process (sample TS-1-a 24/170). The addition of a low-temperature aging stage generally promotes a reduction in crystal size by a factor of two or more, as well as the formation of particles with an elliptical morphology, such as sample TS-1-a 24/60-72/170, with an average particle size of 200–220 nm, and samples TS-1-a 24/60-24/130 and TS-1-a 24/60-24/170, with particle sizes of 150–180 nm. When the crystallization temperature at the second stage decreases from 170 °C to 130 °C, the crystal morphology changes and nanosized flake-like aggregates are actively formed. The use of acid-containing gels during the hydrothermal crystallization process facilitates the formation of smaller crystals (compare samples TS-1-a 24/60-72/170 and TS-1-b 24/60-72/170).

#### 2.1.3. Coordination of Ti Atoms

**Figure 3:**
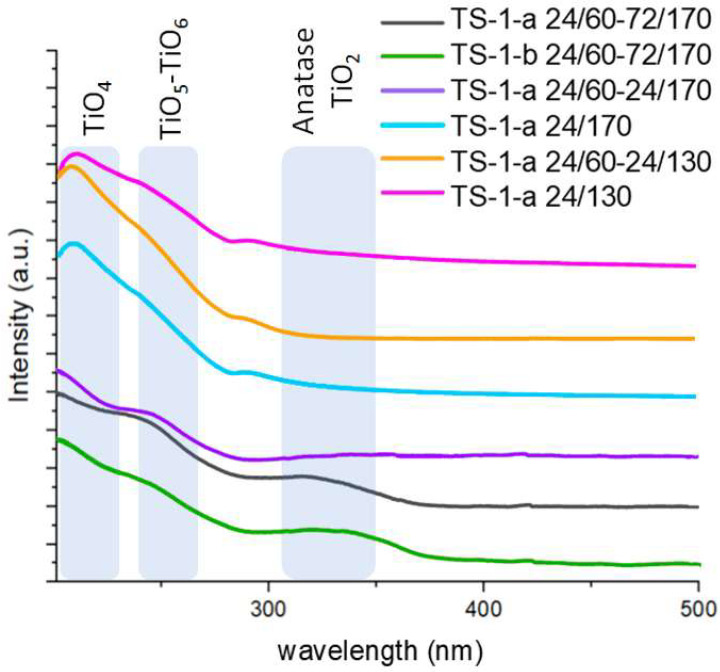
UV–vis spectra of samples obtained from acid- and base-catalyzed gels (TS-1-a and TS-1-b, respectively).

To determine the nature and coordination state of titanium atoms in the structure of crystalline TS-1, as well as the presence of extra-framework TiO_2_ species, DR UV–vis spectroscopy was used (Figure 3).

The spectra of synthesized titanosilicates contain a band centered at 210–220 nm, which corresponds to isolated Ti^4+^ atoms in tetrahedral coordination [26,27]. A broad shoulder in the 250–285 nm range is generally attributed to titanium atoms in higher coordination states, such as penta-, hexa- [28], or octacoordination [29]. The absorption band at 310–350 nm, indicative of the presence of extra-framework anatase TiO_2_ phase [30], is observed only in samples TS-1-b 24/60-72/170 and TS-1-a 24/60-72/170. This band is absent in all other samples, indicating the absence of TiO_2_ phase formation.

Thus, when synthesizing TS-1 titanosilicate via the sol-gel approach, the crystallization conditions play a crucial role in obtaining titanosilicates free of extra-framework TiO_2_. It should be noted that the extra-framework TiO_2_ phase is observed in samples synthesized under the “harshest” conditions: two crystallization stages, the maximum duration (96 h), and the highest temperature at the second stage (170 °C). Using an acid-containing gel as the precursor and reducing the crystallization time to 24 h favor the formation of TS-1 titanosilicate samples in which titanium is incorporated into the zeolite framework. The highest content of penta- and hexacoordinated titanium is promoted by the combination of the acidic stabilization of titanium during synthesis and the subsequent crystallization at the highest temperature.

#### 2.1.4. Textural Properties

**Figure 4:**
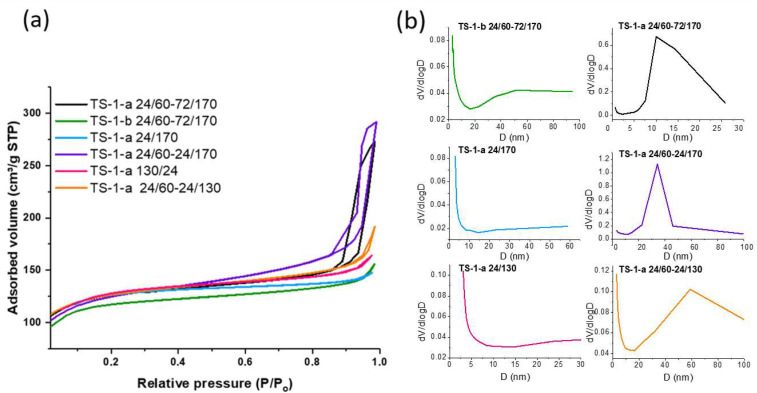
(**a**) N_2_ adsorption and desorption isotherms, (**b**) pore size distribution of obtained TS-1-b and TS-1-a samples.

Changes in the textural characteristics of the synthesized titanosilicates were studied using low-temperature N_2_ adsorption–desorption (Table 1, Figure 4).

Analysis of the textural parameters shows that the TS-1-b sample is a microporous material and has an adsorption isotherm shape typical of microporous materials and a micropore volume (0.16 cm^3^/g) characteristic of the MFI structure. The small amount of mesopores (0.04 cm^3^/g) in the sample is attributed to intercrystalline voids. The TS-1-a series samples obtained using acid at the stage of obtaining the precursor gel are characterized by both micro- and micro-mesoporous structures. The titanosilicate samples obtained by single-stage crystallization (TS-1-a 24/130, TS-1-a 24/170) are close in terms of characteristics to the TS-1-b sample. These are predominantly microporous materials; the mesopore volume in them is insignificant. Samples obtained via two-step crystallization exhibit mesoporosity, as evidenced by the appearance of a hysteresis loop on the adsorption isotherm. The hysteresis loop is relatively small for the TS-1-a 24/60-24/130 sample, synthesized under the mildest crystallization conditions, and increases with higher crystallization temperatures and prolonged crystallization times. A sharp rise in the loop in the relative pressure range of 0.8 < P/P_0_ < 1.0 indicates the existence of well-designed mesopores [31].

The mesopore volume ranges from 47% (TS-1-a 24/60-24/130) to 71% (TS-1-a 24/60-72/170) of the total pore volume. The samples with the smallest crystallite size (TS-1-a 24/60-24/130 and TS-1-a 24/60-24/170) show the largest SBET values and outer surface values comparable to the single-stage TS-1.

Heteroatomic zeolites, like zeolites themselves, are microporous [32]. Mesoporosity can be formed by destructive methods (alkali or acid treatment) and by direct synthesis using a mesoporous template (intracrystalline mesoporosity), or as a result of aggregation of the small crystals during crystallization (intercrystalline mesoporosity). The nature of mesoporosity for the synthesized samples is intercrystalline pore formation, which is often the case for materials with crystallites of less than 200 nm obtained without mesoporous templates or special post-treatments [33]. This result is consistent with the SEM findings (Figure 2). Analysis of the pore size values shows that the TS-1-a 24/60-24/130, TS-1-a 24/60-24/170, and TS-1-a 24/60-72/170 samples demonstrated a hysteresis loop (Figure 4a) and exhibited a monomodal distribution (Figure 4b). The narrowest BJH pore size distribution centered at 34 nm can be observed for the TS-1-a 24/60-24/170 sample, which also possessed the highest pore volume. The sample synthesized under the milder crystallization conditions (TS-1-a 24/60-24/130) showed a combination predominantly consisting of micro- and macropores. This is further supported by the nature of the N_2_ adsorption–desorption isotherm, namely, the presence of a very narrow hysteresis loop and a sharp increase at high relative pressure. For samples obtained using only TPAOH or using acid, however, without the aging step, the predominant presence of micropores and some macropores was confirmed.

Thus, the use of acid as a catalyst for hydrolysis at the stage involving precursor gel production, as well as the two-step crystallization of the precursor gel with an increase in temperature during the second step, results in the creation of TS-1 titanosilicates with a hierarchical micro-mesoporous surface.

#### 2.1.5. FTIR Study of Concentration of Brønsted and Lewis Acid Sites and Hydroxyl Group Bands

**Figure 5:**
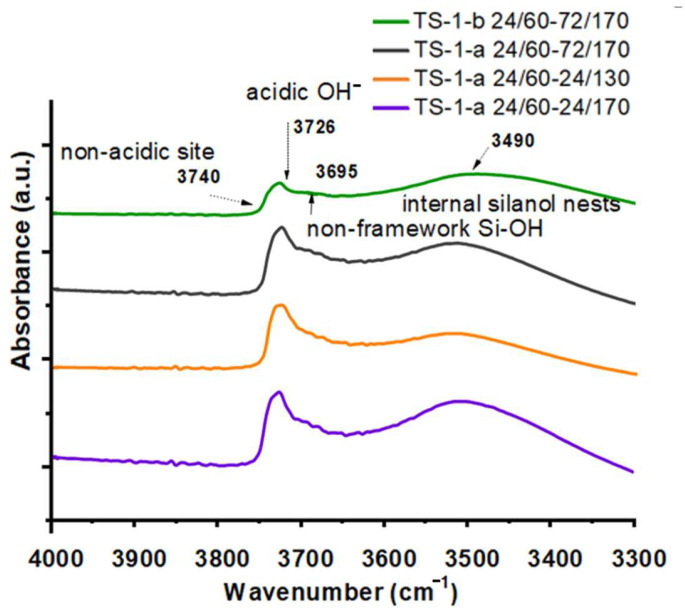
FTIR spectra of samples synthesized from acid- and base-catalyzed gels (TS-1-a and TS-1-b, respectively).

The nature and distribution of hydroxyl groups in several samples were studied (Figure 5). The samples were selected based on two criteria: (a) the effect of the hydrolysis agent on the early stages of synthesis, and (b) comparison with samples containing crystallites smaller than 200 nm and free from non-framework Ti.

In the IR spectra of the studied samples, in the vibration range of 4000–3300 cm^−1^ (Figure 5), vibration bands typical of OH groups are observed for titanosilicate TS-1 [34]. The bands at 3740 cm^−1^ and 3726 cm^−1^ characterize vicinal internal (SiO)_3_Si-OH-OH-Si(SiO)_3_ groups and/or (SiO)_3_Si-OH-OH-Ti(SiO)_3_ in framework defects, as well as isolated (SiO)_3_Si-OH and/or (SiO)_3_Ti-OH groups located on the outer surfaces of the crystals. The broad band with a maximum of about 3480 cm^−1^ is due to vibrations of the internal silanol groups Si-OH bonded by hydrogen in internal silanol nests, which are defective areas in the crystals. The formation of a greater number of defects in the TS-1-a 24/60-72/170 and TS-1-a 24/60-24/170 crystals is apparently associated with the conditions of their synthesis. Crystallization at a high temperature of 170 °C leads to more noticeable destruction of the crystal lattice, as evidenced by the high concentration of internal hydroxyls in the silanol nests. At the same time, in the spectrum of the TS-1-a 24/60-24/130 sample, the intensity of the band in the region of 3480 cm^−1^ is lower; accordingly, lowering the temperature of the second stage of crystallization to 130 °C leads to the acquirement of a material with a smaller number of defects. The high intensity of the band at 3726 cm^−1^ for samples TS-1-a 24/60-72/170, TS-1-a 24/60-24/130, and TS-1-a 24/60-24/170 compared to TS-1-b 24/60-72/170 indicates a high concentration of isolated hydroxyl groups in the first three samples, which is due to their smaller crystal size.

**Figure 6:**
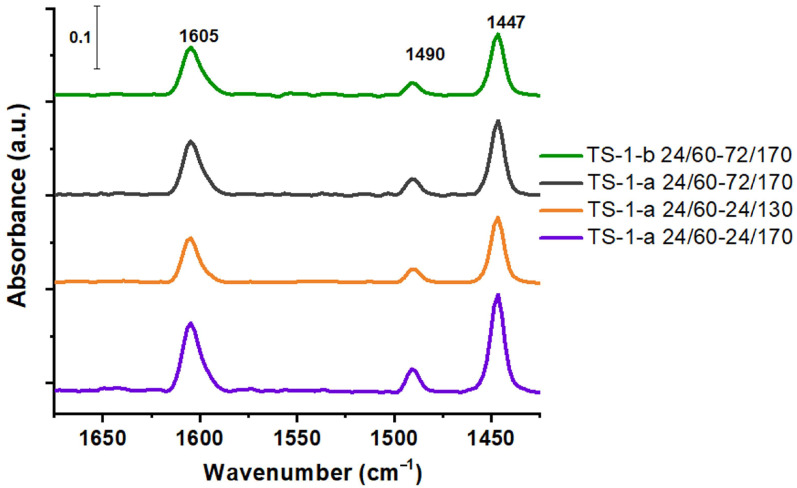
FTIR spectra of pyridine adsorbed on TS-1 samples prepared from acid- and base-catalyzed gels (TS-1-a and TS-1-b, respectively).

When studying the acidic properties of the samples synthesized by means of two-stage crystallization using FTIR spectroscopy with pyridine adsorption (Figure 6), the presence of Lewis acid sites (LAC) on the surface of the obtained titanosilicates was established, as evidenced by the presence of absorption bands in the FTIR spectrum at 1605, 1447 cm^−1^ (corresponding to pyridine that formed a coordination bond with the LAC). The absorption band in the region of 1490 cm^−1^ appears as a result of the interaction of pyridine with Lewis and Brønsted acid sites. The source of the strong Lewis acid sites could be coordinatively unsaturated and saturated titanium ions in the framework [35].

In titanosilicates, the active sites (usually four coordinated titanium atoms) are thought to act as weak Lewis acids. When H_2_O_2_ is used as an oxidizing agent, the titanium atom pulls electrons away from the peroxide, thereby reducing the electron density of the O-O bond. As a result, the peroxide becomes more susceptible to nucleophilic attack by the alkene.

The shoulder at 1598 cm^−1^ indicates the presence of a small amount of pyridine, which formed a weak hydrogen bond with the OH groups. The presence of Brønsted acid sites (BAS) in the TS-1 structure is associated with the presence of silanol and titanol groups [34]. After calcination at 200 °C, the concentration of the adsorbed pyridine decreased sharply. The very low content of BAS indicates an insignificant amount of Ti-OH groups in the obtained materials, formed during the hydrolysis of the Ti-O-Si bond. The concentration of pyridine after desorption at 150 °C (Table 2) changed in the following series: TS-1-a 24/60-24/130 < TS-1-b 24/60-72/170 < TS-1-a 24/60-72/170 < TS-1-a 24/60-24/170. Sample TS-1-a 24/60-24/130 is characterized by the smallest number of defects in the structure and has the lowest concentration of strong LAS (based on data obtained during the desorption of pyridine at 250 °C)—4 μmol/g. In contrast, sample TS-1-a 24/60-24/170 has the highest concentration of strong LASs—15 μmol/g (desorption at 250 °C).

Thus, the use of the described strategy, including (a) using acid as a catalyst for the hydrolysis of the silicon source at the first stage of obtaining the precursor gel; and (b) carrying out the crystallization process in two stages, first at 60 °C and then at 130 or 170 °C, leads to the production of TS-1 titanosilicates with a hierarchical (micro-meso) porous surface and a crystal size of 150–550 nm, containing titanium in the form of TiO_4_ and TiO_6_ and no extra-framework TiO_2_, and also possessing Lewis acidity.

#### 2.1.6. Catalytic Performance in Epoxidation of Cyclohexene

The catalytic properties (activity and selectivity) of the synthesized titanosilicate samples were studied in a model reaction of cyclohexene epoxidation with hydrogen peroxide. The choice of this reaction was due to the fact that, as indicated in [36], cyclohexene is a very sensitive model substrate for testing the activity and selectivity of epoxidation reaction catalysts. The size of the cyclohexene molecule is about 0.5 nm, i.e., close to the size of the inlet windows in the MFI zeolite channels (0.51–0.55 nm), which can create significant diffusion difficulties during its movement in the channels to the active centers. Diffusion of the reaction products from the zeolite channels into the reaction volume is also difficult. In addition, the product of cyclohexene epoxidation reaction, 1,2-epoxycyclohexane, is prone to ring-opening reactions, since its epoxide ring is highly strained [37]. According to the data given in [36] and according to the results of cyclohexene epoxidation with hydrogen peroxide on titanosilicate catalysts in the presence of traditional TS-1 titanosilicates with a microporous structure, the conversion of cyclohexene varies in the range of 0.2–26.3%, and the selectivity for the formation of 1,2-epoxycyclohexane can be from 22 to 75% when the reaction is carried out under similar conditions (60 °C, 2–4 h, acetonitrile solvent).

The results obtained are presented in Table 3.

Under the studied conditions (60 °C, 5 h, AcCN, cyclohexene 10 mmol, H_2_O_2_ 10 mmol, 0,1 g catalyst), all the obtained catalysts showed activity in the cyclohexene oxidation reaction. The maximum conversion of cyclohexene (15%) was achieved by the TS-1-a 24/60-72/170 sample. For the other titanosilicates, the cyclohexene conversion value was 6–10%. In general, it can be noted that for the microporous samples (TS-1-b 24/60-72/170, TS-1-a 24/130, TS-1-a 24/170), the cyclohexene conversion was somewhat lower than that of the hierarchical ones, which is apparently due to the reaction occurring mainly on the surface active centers. Among the samples with a hierarchical porous structure, titanium silicate TS-1-a 24/60-72/170 stands out, demonstrating the highest activity, which is apparently due to its possession of the most developed mesoporosity (Table 1). Note that this sample contains an anatase phase, which, we assume, can also contribute to the catalytic activity, although some authors [38] believe that anatase is not active in the oxidation reaction, but only decomposes hydrogen peroxide.

**Figure 7:**
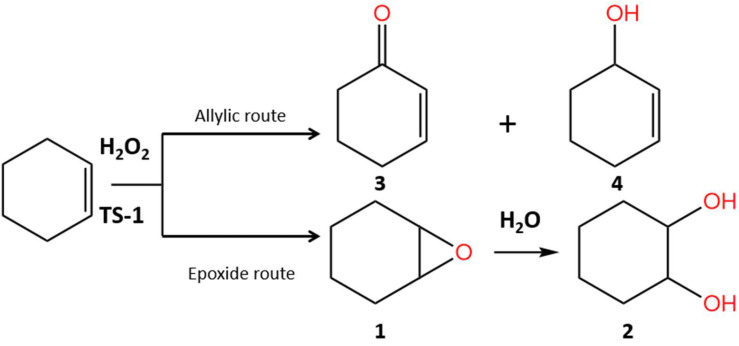
Oxidation of cyclohexene on surfaces of TS-1 titanosilicates: epoxycyclohexane (**1**), cyclohexane-1,2-diol (**2**), 2-cyclohexen-1-one (**3**), 2-cyclohexen-1-ol (**4**).

Based on the composition of the reaction mass formed on the synthesized titanosilicates, the oxidation of cyclohexene proceeds in two directions (Figure 7).

Via the epoxide route, epoxycyclohexane (**1**) and cyclohexane-1,2-diol (**2**) are formed. Via the allylic pathway, 2-cyclohexen-1-one (enone, **3**) and 2-cyclohexen-1-ol (enol, **4**) are produced. The selectivity toward these compounds strongly depends on the catalyst used (Table 3). The highest selectivity for epoxidation products is observed for the TS-1-a 24/60-24/170 and TS-1-a 24/60-24/130 samples, where the selectivity for epoxide **1** reaches 48% and 57%, respectively. Both samples are characterized by a hierarchical porous structure, small crystal size, and the absence of an extra-framework anatase phase. The difference in the selectivity of the action of these samples can apparently be explained by several factors. One of them is the differences in acidity: the TS-1-a 24/60-24/130 sample, on which epoxide 1 is formed most selectively, contains a smaller amount of LAS compared to titanosilicate TS-1-a 24/60-24/170, including a minimal amount of strong LAS (4 μmol pyridine/g). An increase in the number of LAS, including strong Lewis centers (15 μmol pyridine/g), as observed on the TS-1-a 24/60-24/170 sample, promotes the intensification of reactions occurring via the allylic route. In this case, the intermediate peroxide compounds formed with titanium atoms interact with the allyl bond -C–H, resulting in the formation of enone **3** and enol **4**.

Another factor is the presence or absence of defects in the titanosilicate structure. The most selective sample, TS-1-a 24/60-24/130, is characterized by a minimal number of structural defects (Figure 5), which may contribute to its high selectivity in the formation of 1,2-epoxycyclohexane. Literature reports indicate that on titanosilicates with hierarchical porous structures, the epoxy ring-opening reaction is significantly enhanced. It is important to emphasize that the hierarchically structured titanosilicates synthesized in this work are free from this drawback. The selectivity for diol formation in samples TS-1-a 24/60-24/130 and TS-1-a 24/60-72/170 is 3% and 7%, respectively, while no diol was detected in the reaction products of sample TS-1-a 24/60-24/170.

Similar selectivity toward the products is observed for the TS-1b 24/60-72/170 and TS-1a 24/60-72/170 samples. These samples were obtained under the harshest conditions and differ in terms of the gel preparation method. It should be noted that the first sample is microporous, while the second is micro-mesoporous. In both samples, the products formed via the allylic route—enone **3** and enol **4**—predominate, with a combined selectivity of 60% and 57%, respectively. An anatase phase is present in both samples.

In the presence of samples obtained by one-step crystallization (TS-1-a 24/130 and TS-1-a 24/170), a comparable ratio of allylic oxidation products (**3**) and (**4**) and epoxidation products (**1**) and (**2**) is observed in the reaction mixture.

Thus, in the epoxidation of cyclohexene, the highest selectivity was demonstrated by titanosilicate samples TS-1-a 24/60-24/130 and TS-1-a 24/60-24/170, which possess a hierarchical porous structure, contain Lewis acid sites, and are free of TiO_2_ phase. Samples with a microporous structure (TS-1-b 24/60-72/170, TS-1-a 24/130, TS-1-a 24/170) tend to catalyze allylic oxidation reactions, as does the hierarchical titanosilicate TS-1-a 24/60-72/170, which contains an anatase phase within its structure.

## 3. Conclusions

A new simple approach is proposed to synthesize nanosized (150–180 nm) hierarchical anatase-free TS-1 titanosilicates. This involves obtaining a precursor gel using an acid catalyst. The principal differences between this approach and other methods using acids are that nitric acid is introduced in small quantities (molar ratio Si/H^+^ = 120/1) at the initial stage of tetraethyl orthosilicate hydrolysis and the introduction of titanium isopropoxide solution occurs in the presence of acid protons. The gel is formed by a sharp change in pH to 12. To control the textural parameters, it is necessary to vary the conditions of the subsequent crystallization of the resulting mixture. The preliminary aging stage allows one to directly obtain nanosized (150–180 nm) and hierarchical TS-1 titanosilicates upon crystallization at 130–170 °C for 24 h (TS-1 a 24/60-24/130 and TS-1 a 24/60-24/170). At higher crystallization temperatures, the proportion of mesopores in the TS-1 a 24/60-24/170 sample is 67% and the highest concentration of LAS is obtained. A threefold increase in the crystallization time leads to an increase in the proportion of mesopores to 71% (TS-1 a 24/60-72/170) and a crystallite size of 200–220 nm, as well as the formation of extra-framework titanium. When the hydrolysis catalyst is changed from acidic to basic during the formation of the precursor gel, the TS-1 b 24/60-72/170 sample is obtained, which is predominantly microporous and has crystallites up to 370 nm, extra-framework titanium, and the lowest LAS content.

The activity and selectivity of the synthesized titanosilicates during the oxidation of cyclohexene with hydrogen peroxide were studied. It was shown that under the studied conditions (60 °C, cyclohexene 10 mmol, H_2_O_2_ 10 mmol, 0.1 g catalyst, 5 h, AcCN) the reaction proceeds along one of two routes: via the epoxy route with the formation of 1,2-epoxycyclohexane (**1**) and cyclohexane-1,2-diol (**2**), or via the allyl route with the formation of 2-cyclohexen-1-one (**3**) and 2-cyclohexen-1-ol (**4**). The formation of epoxy compound **1** is most favored by the titanosilicates TS-1-a 24/60-24/130 and TS-1-a 24/60-24/170, which have a hierarchical porous structure, the smallest crystals, and Lewis acid sites, and lack the TiO_2_ phase. The maximum selectivity (67%) at a cyclohexene conversion of 10% was achieved by the TS-1-a 24/60-24/130 sample. The presence of an anatase phase in the hierarchical titanosilicate (sample TS-1a 24/60-72/170) leads to an increase in catalyst activity and a change in its catalytic action: oxidation occurs predominantly along the allylic route with the formation of ketone (**3**) and alcohol (**4**) (total selectivity is 57% at a conversion of 15%). The microporous titanosilicates TS-1-b 24/60-72/170, TS-1-a 24/130, and TS-1-a 24/170 are somewhat less active than the hierarchical ones and predominantly catalyze the reaction of allylic oxidation of cyclohexene.

The synthesized nanosized hierarchical TS-1 titanosilicates can be employed in the oxidation of various bulky molecules, including cycloolefins and phenols, whose epoxidation is inefficient in the presence of conventional microporous titanosilicates. These catalysts will enable oxidation via a “green” method, using hydrogen peroxide as the oxidant.

## 4. Materials and Methods

### 4.1. Synthesis of Titanosilicates TS-1 via Gels Formation

The following were used for the synthesis of catalysts: tetraethyl orthosilicate (TEOS, analytical grade, Bashkhimservice, Ufa, Russia), titanium isopropoxide (97%, Sigma-Aldrich, St. Louis, MO, USA), isopropyl alcohol (chemically pure, Bashkhimservice), and nitric acid (chemically pure, Bashkhimservice).

Titanosilicates TS-1 were synthesized using the following method. Initially, precursor gels were prepared, which were subsequently subjected to hydrothermal treatment. The initial precursor gels differed in terms of their synthesis method. In the synthesis of the first type, the hydrolysis of the silicon source was catalyzed by introducing a part of the TPAOH solution with the OH^−^/Si = 1/120 (sample TS-1-b). For the second type of gel (samples TS-1-a), the hydrolysis of the silicon source was accelerated by introducing a nitric acid solution taken in a molar ratio of H^+^/Si = 1/120.

The molar composition of the gels is SiO_2_: 0.025 TiO_2_: 0.38 TPAOH: 48 H_2_O: 0.008X, where X is either an additional amount of TPAOH or HNO_3_, introduced at the first step of the synthesis. To obtain the gel, firstly, TEOS is mixed with water and a hydrolysis catalyst. Once a homogeneous solution is formed, an isopropanol solution of titanium isopropoxide is added (7 mL of solvent to 0.22 g of Ti source). The final solution is kept under stirring for up to 15 h. Then the gel is formed by dropwise introduction of the TPAOH solution.

The resulting mixture is stirred until homogeneity is reached. After, it is transferred to the autoclaves (Teflon-lined steel autoclave) and the aging stage begins at 60 °C. Then it is crystallized at 170 or 130 °C. For samples obtained without preliminary heat treatment, the aging stage is skipped. Depending on the crystallization temperature and holding time, the samples are designated TS-1-a (t1/T_1_-t2/T_2_), where T_1_ and T_2_ are the crystallization temperature, and t1 and t2 are the processing time in hours at the aging and crystallization stages, respectively. The obtained samples are centrifuged, washed with distilled water, then dried at room temperature and calcined at 550 °C for 2 h.

### 4.2. Catalyst Characterization

Morphological characterization was conducted using a Hitachi Regulus SU8220 scanning electron microscope (SEM) (Hitachi High-Technologies, Tokyo, Japan), which is equipped with a secondary electron (SE) detector employing a photomultiplier, as well as a bright-field scanning transmission electron microscopy (BF-STEM) detector fitted with an additional diaphragm. Samples were deposited onto a copper grid mounted on an aluminum holder for SEM analysis. Electron micrographs of all four samples were acquired at an accelerating voltage of 30 kV. Additionally, imaging was performed at magnifications of 60,000×, 70,000×, 80,000×, and 100,000×.

The phase composition and crystallinity of the samples were assessed using X-ray diffraction (XRD) analysis through a Shimadzu XRD-7000 diffractometer (Shimadzu Corp., Tokyo, Japan) with monochromated CuKα radiation over a 2θ range from 5° to 40°, at a scanning speed of 0.5 degrees per minute. The diffraction patterns of TS-1 were analyzed by comparison with the PDF2 powder diffraction database and further corroborated using the IZA database.

The specific surface area and the volume of micro- and mesopores were examined by the low-temperature (−196 °C) adsorption–desorption of N_2_ technique with a NOVA 1200 e (Quantachrome, Boynton Beach, FL, USA) sorption meter (Quantachrome, Boynton Beach, FL, USA). Before the analysis, titanosilicate samples were treated under a vacuum at 350 °C for 4 h. The specific surface area was determined by the BET method at a relative partial pressure P/P_0_ = 0.2. Micropore volume was calculated by the t-plot method. Pore size distribution was calculated by the Barrett–Joyner–Halenda (BJH) method from the desorption curve.

The states of titanium in the samples were determined by UV–vis diffuse reflectance spectra recorded on a TUV10DCS spectrometer (Persee, Beijing, China) equipped with deuterium, tungsten, and IR lamps. The spectrometer was equipped with an integrating sphere. The standard white plate from BaSO_4_ was used to measure reflectance. The spectrum was recorded in the range of 200–600 nm.

The acidic properties of the TS-1 samples were investigated using FTIR spectroscopy with a pyridine probe molecule (Py-FTIR). All spectra were recorded with a Bruker Vertex-70 V FTIR spectrometer (Bruker, Karlsruhe, Germany) at a resolution 4 cm^−1^ in the range 400–4000 cm^−1^. The weight-to-diameter ratio of the pellet used for recording the IR spectra was 10 mg/cm^2^. Prior to analysis, the applied samples were heat-treated at 450 °C in a vacuum (0.5 Pa) for 2 h. The pyridine adsorption was performed at 150 °C and a pressure of 300 Pa for 30 min, after which the sample was evacuated at 150 °C for 30 min. The IR spectra were recorded at room temperature before adsorption and immediately after the pyridine desorption at 150 °C. The LAS quantity was calculated by integration of the peak at 1435–1470 cm^−1^, using the known integral molar extinction coefficients of pyridine for these sites [39].

### 4.3. Catalytic Performance

In this work, cyclohexene (Cy) (99.0%, Sigma-Aldrich), acetonitrile (99.9%, Ecos-1), and hydrogen peroxide 30% (OST 301-02-205-99) were used.

Catalytic studies were carried out in a glass flask with temperature control and a reflux condenser, with stirring and controlled heating for 5 h at 60 °C and at atmospheric pressure. The molar ratio of cyclohexene:H_2_O_2_ = 10:10 mmol/mmol, the solvent acetonitrile—5 mL. The mass of the catalyst was 100 mg. The reaction mass was analyzed by GLC on a Chromatec Crystal 5000 chromatograph (Chromatec SDO JSC, Yoshkar- Ola, Russia, packed column 3 m; phase 5% PEG—6000; carrier gas—helium; temperature programming 50–270 °C; detector—FID).

The conversion of reactants (X, %) and the selectivity of product formation (S_i_, %) were calculated as follows:

X (%) = 100% × (C_Cy in feed_ − C_Cy in reaction mixture_)/C_Cy in feed_ (1)

where C_Cy in feed_ and C_Cy in reaction mixture_ are the cyclohexene concentrations before and after the reaction, wt%;

S_i_ (%) = 100% × C_i_/ΣC_i_, (2)

where C_i_ is the concentration of the ith product in the reaction mixture, wt%; ΣC_i_ is the total concentration of all products, wt%.

The material balance for each of the experiments was greater than 95%.

Relative response factors were determined using synthetic mixtures of cyclohexene and the main products. Product identification and confirmation were performed by gas chromatography–mass spectrometry (GC-MS) through a Shimadzu GCMS-QP2010 quadrupole instrument with electron ionization (70 eV). The following were used: a scanning range of 10–350 m/z; range scanning time of 0.22 s; ion source temperature of 200 °C; chromatograph injector temperature of 250 °C; interface temperature of 250 °C; flow split from 1:40 to 1:120; ZB-5MS capillary column (Phenomenex, Torrance, CA, USA) measuring 30 m × 0.25 mm × 0.25 μm; helium carrier gas, at a flow rate of 0.8 mL/min; thermostat temperature of 40 °C–1 min; heating at 12 °C/min to 110 °C, 110 °C–3 min; heating at 15 °C/min to 280 °C, 280 °C–10 min.

## Figures and Tables

**Table 1:** Textural characteristics of studied TS-1 samples.

Sample	S_BET_ (m^2^/g)	V_micro_ (cm^3^/g)	V_meso_ (cm^3^/g)	V_total_ (cm^3^/g)
TS-1-b 24/60-72/170	350	0.15	0.04	0.19
TS-1-a 24/60-72/170	406	0.12	0.30	0.42
TS-1-a 24/60-24/170	461	0.15	0.30	0.45
TS-1-a 24/60-24/130	464	0.16	0.14	0.3
TS-1-a 24/130	394	0.17	0.08	0.25
TS-1-a 24/170	380	0.18	0.05	0.23

**Table 2:** Acidic properties of titanosilicate samples according to FTIR spectroscopy of adsorbed pyridine.

Sample	Concentration of Acid Sites, µmol Py/g		
	LAS		
	150 °C	200 °C	250 °C
TS-1-b 24/60-72/170	145	33	9
TS-1-a 24/60-72/170	172	31	8
TS-1-a 24/60-24/130	137	23	4
TS-1-a 24/60-24/170	214	48	15

**Table 3:** Catalytic properties of titanosilicates in oxidation of cyclohexene with hydrogen peroxide (reaction conditions: 60 °C, cyclohexene 10 mmol, H_2_O_2_ 10 mmol, 0,1 g catalyst, 5 h, AcCN).

Sample	Conversion Cyclohexene, %	Selectivity, %			
		Epoxy 1	Enol + Enon 3 + 4	Dio l2	Others
TS-1b 24/60-72/170	7	26	60	3	11
TS-1a 24/130	6	41	42	8	9
TS-1a 24/170	8	37	50	4	9
TS-1a 24/60-72/170	15	26	57	7	10
TS-1a 24/60-24/170	9	48	40	-	12
TS-1a 24/60-24/130	10	67	23	3	7

## Data Availability

The original contributions presented in the study are included in the article, further inquiries can be directed to the corresponding author.

## Abbreviations

The following abbreviations are used in this manuscript:

HPPO	Hydrogen Peroxide to Propylene Oxide
Cy	Cyclohexene
epoxy	epoxycyclohexane
diol	cyclohexane-1,2-diol
enon	2-cyclohexen-1-one
enol	2-cyclohexen-1-ol
LAS	Lewis acid sites
BAS	Brønsted acid sites
FTIR	Fourier transform infrared spectroscopy
DR UV–vis spectroscopy	Ultra-violet–visible diffuse reflectance spectroscopy
BJH method	Barrett–Joyner–Halenda method

## References

[1] Millini R., Previde Massara E., Perego G., Bellussi G. (1992). Framework Composition of Titanium Silicalite-1. J. Catal..

[2] Schmidt F., Bernhard M., Morell H., Pascaly M. (2014). HPPO Process Technology A Novel Route to Propylene Oxide without Coproducts. Chim. Oggi/Chem. Today.

[3] Our Proprietary Catalyst Technology. https://versalis.eni.com/en-IT/portfolio/technologies-licensing/catalyst-technology.html.

[4] Perego C., Carati A., Ingallina P., Mantegazza M.A., Bellussi G. (2001). Production of Titanium Containing Molecular Sieves and Their Application in Catalysis. Appl. Catal. A Gen..

[5] Luan H., Xu C., Wu Q., Xiao F.-S. (2022). Recent Advances in the Synthesis of TS-1 Zeolite. Front. Chem..

[6] Mintova S., Grand J., Valtchev V. (2016). Nanosized Zeolites: Quo Vadis?. Comptes Rendus Chim..

[7] Rodionova L.I., Knyazeva E.E., Konnov S.V., Ivanova I.I. (2019). Application of Nanosized Zeolites in Petroleum Chemistry: Synthesis and Catalytic Properties (Review). Pet. Chem..

[8] Smeets V., Gaigneaux E.M., Debecker D.P. (2022). Titanosilicate Epoxidation Catalysts: A Review of Challenges and Opportunities. ChemCatChem.

[9] Fu G., Dib E., Lang Q., Zhao H., Wang S., Ding R., Yang X., Valtchev V. (2022). Acidic Medium Synthesis of Zeolites—An Avenue to Control the Structure-Directing Power of Organic Templates. Dalton Trans..

[10] Pope E.J.A., Mackenzie J.D. (1986). Sol-Gel Processing of Silica. J. Non-Cryst. Solids.

[11] Ismagilov Z.R., Tsikoza L.T., Shikina N.V., Zarytova V.F., Zinoviev V.V., Zagrebelnyi S.N. (2009). Synthesis and Stabilization of Nano-Sized Titanium Dioxide. Russ. Chem. Rev..

[12] Antonenko M.V., Ogurtsova Y.N., Strokova V.V., Gubareva E.N., Klyuev S.V., Lesovik V.S., Vatin N.I. (2021). The Effect of Titanium Dioxide Sol Stabilizer on the Properties of Photocatalytic Composite Material. Innovations and Technologies in Construction.

[13] Watson S.S., Beydoun D., Scott J.A., Amal R. (2003). The Effect of Preparation Method on the Photoactivity of Crystalline Titanium Dioxide Particles. Chem. Eng. J..

[14] Timofeeva M.N., Kalashnikova G.O., Shefer K.I., Mel’gunova E.A., Panchenko V.N., Nikolaev A.I., Gil A. (2020). Effect of the Acid Activation on a Layered Titanosilicate AM-4: The Fine-Tuning of Structural and Physicochemical Properties. Appl. Clay Sci..

[15] Llabrés i Xamena F.X., Calza P., Lamberti C., Prestipino C., Damin A., Bordiga S., Pelizzetti E., Zecchina A. (2003). Enhancement of the ETS-10 Titanosilicate Activity in the Shape-Selective Photocatalytic Degradation of Large Aromatic Molecules by Controlled Defect Production. J. Am. Chem. Soc..

[16] Lee S.L., Nur H., Wei S.C. (2012). Effect of Acid Treatment on Silica-Titania Aerogel as Oxidative-Acidic Bifunctional Catalyst. Appl. Mech. Mater..

[17] Xiong G., Jia Q., Cao Y., Liu L., Guo Z. (2017). The Effect of Acid Treatment on the Active Sites and Reaction Intermediates of the Low-Cost TS-1 in Propylene Epoxidation. RSC Adv..

[18] Xu H., Zhang Y., Wu H., Liu Y., Li X., Jiang J., He M., Wu P. (2011). Postsynthesis of Mesoporous MOR-Type Titanosilicate and Its Unique Catalytic Properties in Liquid-Phase Oxidations. J. Catal..

[19] Wang Y., Yao Q., Xu B., Lin K., Deng M., Lu X., Ma R., Fu Y., Zhu W. (2023). Ti-MWW Synthesis via Acid-Modulated Isomorphous Substitution of ERB-1 for Efficient Ethylene Oxidative Hydration to Ethylene Glycol. J. Catal..

[20] Wang Y., Li L., Bai R., Gao S., Feng Z., Zhang Q., Yu J. (2021). Amino Acid-Assisted Synthesis of TS-1 Zeolites Containing Highly Catalytically Active TiO6 Species. Chin. J. Catal..

[21] Li M., Shen X., Liu M., Lu J. (2021). Synthesis TS-1 Nanozelites via L-Lysine Assisted Route for Hydroxylation of Benzene. Mol. Catal..

[22] Tyablikov I.A. (2019). Synthesis and Physicochemical Properties of Titanosilicate with MFI Structure as a Catalyst for Epoxidation of Alkenes. Ph.D. Thesis.

[23] Grieneisen J.L., Kessler H., Fache E., Le Govic A.M. (2000). Synthesis of TS-1 in Fluoride Medium. A New Way to a Cheap and Efficient Catalyst for Phenol Hydroxylation. Microporous Mesoporous Mater..

[24] Zhang J., Shi H., Song Y., Xu W., Meng X., Li J. (2021). High-Efficiency Synthesis of Enhanced-Titanium and Anatase-Free TS-1 Zeolite by Using a Crystallization Modifier. Inorg. Chem. Front..

[25] Perera A.S., Trogadas P., Nigra M.M., Yu H., Coppens M.-O. (2018). Optimization of Mesoporous Titanosilicate Catalysts for Cyclohexene Epoxidation via Statistically Guided Synthesis. J. Mater. Sci..

[26] Geobaldo F., Bordiga S., Zecchina A., Giamello E., Leofanti G., Petrini G. (1992). DRS UV-Vis and EPR Spectroscopy of Hydroperoxo and Superoxo Complexes in Titanium Silicalite. Catal. Lett..

[27] Serrano D.P., Sanz R., Pizarro P., Moreno I. (2012). Tailoring the Properties of Hierarchical TS-1 Zeolite Synthesized from Silanized Protozeolitic Units. Appl. Catal. A Gen..

[28] Guo T., Wang B., Xue C., Liu X., Lin C., Xie X., Luo Y., Liao W., Shu X. (2024). In Situ Tailoring the Crystalline Defects of Titanium Silicalite-1 (TS-1) to Improve the 1-Butene Epoxidation Performance. Ind. Eng. Chem. Res..

[29] Li N., Chen R., Miao J., Zhou P., Yu H.-B., Chen T.-H. (2015). Synthesis of Single Crystal-like Hierarchically Mesoporous Titanosilicate Ti-SBA-1. Chin. Chem. Lett..

[30] Carati A., Flego C., Previde Massara E., Millini R., Carluccio L., Parker W.O., Bellussi G. (1999). Stability of Ti in MFI and Beta structures: A comparative study. Microporous Mesoporous Mater..

[31] Hou W., Lin K., Zhang X., Xu B., Wang Y., Lu X., Gao Y., Ma R., Fu Y., Zhu W. (2023). Highly Stable and Selective Pt/TS-1 Catalysts for the Efficient Nonoxidative Dehydrogenation of Propane. Chem. Eng. J..

[32] Martínez C., Corma A. (2011). Inorganic Molecular Sieves: Preparation, Modification and Industrial Application in Catalytic Processes. Coord. Chem. Rev..

[33] Tao Y., Kanoh H., Abrams L., Kaneko K. (2006). Mesopore-Modified Zeolites:  Preparation, Characterization, and Applications. Chem. Rev..

[34] Tekla J., Tarach K.A., Olejniczak Z., Girman V., Góra-Marek K. (2016). Effective Hierarchization of TS-1 and Its Catalytic Performance in Cyclohexene Epoxidation. Microporous Mesoporous Mater..

[35] Tang Z., Yu Y., Liu W., Chen Z., Wang R., Liu H., Wu H., Liu Y., He M. (2020). Deboronation-Assisted Construction of Defective Ti(OSi)_3_ OH Species in MWW-Type Titanosilicate and Their Enhanced Catalytic Performance. Catal. Sci. Technol..

[36] Přech J. (2018). Catalytic Performance of Advanced Titanosilicate Selective Oxidation Catalysts—A Review. Catal. Rev..

[37] Na K., Jo C., Kim J., Ahn W.-S., Ryoo R. (2011). MFI Titanosilicate Nanosheets with Single-Unit-Cell Thickness as an Oxidation Catalyst Using Peroxides. ACS Catal..

[38] Tatsumi T. (2009). Metal-Substituted Zeolites as Heterogeneous Oxidation Catalysts. Modern Heterogeneous Oxidation Catalysis: Design, Reactions and Characterization.

[39] Shamzhy M., Přech J., Zhang J., Ruaux V., El-Siblani H., Mintova S. (2020). Quantification of Lewis Acid Sites in 3D and 2D TS-1 Zeolites: FTIR Spectroscopic Study. Catal. Today.

